# EHMTI-0090. Botox in the prevention of chronic migraine; comparing NICE criteria versus hull criteria for evaluating responder rate

**DOI:** 10.1186/1129-2377-15-S1-G2

**Published:** 2014-09-18

**Authors:** H Zafar, M Khalil, F Ahmed

**Affiliations:** 1 Neurology, Hull Royal Infirmary, Hull, UK

## Background

Chronic migraine (CM) affects 2% of the population and Botox is the only licensed treatment for prevention of adult patients with CM.

In the UK, National Institute for Clinical Excellence (NICE) approved its use on the National Health Service (NHS) provided patients had failed three preventive medications and appropriately addressed for medication overuse.

NICE defines responder with 30% reduction in headache days without emphasis on severity of headache or number of migraine days.

We developed Hull Criteria that defines responder as one with either:

50% reduction in either

Headache days

Or migraine days

An increment in crystal clear days twice that of baseline

## Objectives

To compare the outcomes of patients receiving Botox treatment in CM according to NICE and Hull Criteria.

## Method

Adult patients with CM attending the Hull migraine clinic were offered Botox based on clinical needs and maintained a headache diary.

Data were extracted for headache, migraine, and headache-free days

Responder rate was assessed applying Hull and NICE criteria.

## Results

Out of a cohort of 357 patients having received a total of 858 cycles, we analysed 151 patients who had received two treatment cycles as recommended by NICE.

A Significant number of patients who responded with Hull Criteria did not satisfy NICE criteria and were denied treatment.

## Discussion

We recommend that severity of headache and number of migraine days must be taken in to account in evaluating response rate to Botox.

NICE criteria should include reduction in migraine days in addition to headache days.

